# Sudden paraparesis due to spinal cord ischemia with initial contrast enhancement of the cauda equina and time-delayed owl-eyes sign on follow-up MR imaging: a case report

**DOI:** 10.1186/s42466-021-00112-5

**Published:** 2021-04-01

**Authors:** Benedict Breitling, Frederic Carsten Schmeel, Alexander Radbruch, Oliver Kaut

**Affiliations:** 1 Department of Neurology, University Clinic Bonn, Venusberg Campus 1, 53105 Bonn, Germany; 2 Department of Neuroradiology, University Clinic Bonn, Bonn, Germany

**Keywords:** Case report, Spinal cord ischemia, Cauda equina, Contrast enhancement

## Abstract

We report on a case of a 52-year-old male with sudden paraparesis. The initial MRI showed contrast enhancement of the conus medullaris and the complete cauda equina. Follow-up MRI revealed a spinal ischemia in the anterior portion of the spinal cord. Only a few reports with similar findings have been published. We suggest that contrast enhancement of the conus medullaris and descending nerve roots can be a potential first indicator of a spinal cord ischemia.

Accurate diagnosis of spinal cord ischemia is challenging with considerable prognostic implications. We report a case of a 52-year-old male patient with sudden paraparesis who was admitted with the suspected diagnosis of GBS.

Spinal cord ischemia is a rare disease and accounts for less than 1% of all strokes and 6–8% of spinal cord diseases [1–3].

MRI is considered the most sensitive imaging modality for detecting spinal cord ischemia [1, 2]. However, specific MRI findings may often occur late in the course or not at all [1, 2] To date, there are few studies that have demonstrated contrast enhancement of the cauda equina as a result of spinal infarction [1, 4–8].

Our patient presented with sudden sensory disturbances and paresis of both legs (MRC 2/5), which rapidly led to inability to walk and loss of the superficial sensitivity from dermatome L1 downwards. The proprioception, the discrimination of blunt or sharp objects and the thermaesthesia were unaffected.

Reflexes of the lower extremities were absent. The reflexes of the upper extremities were unaffected. Further the patient showed no pyramidal signs. He also developed a urinary retention and a constipation of stool.

On the day of admission (2 h after symptom onset), MRI findings showed contrast enhancement of the conus medullaris and the anteriorly as well as the posteriorly located spinal nerve roots extending beyond the spinal canal into the lumbosacral plexus (Fig. 1).

**Fig. 1:**
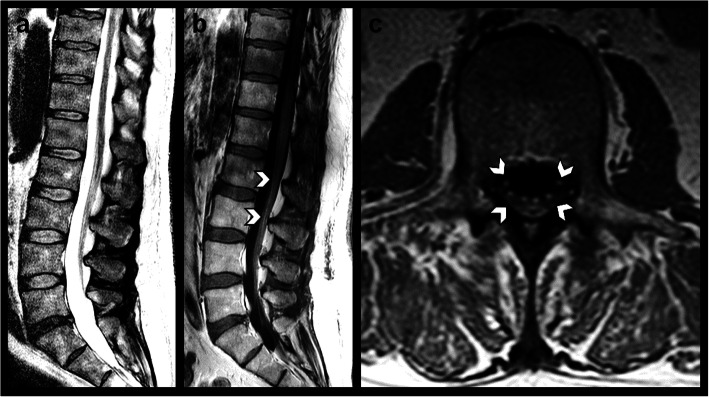
Contrast-enhanced whole-spine MRI performed 2 h after symptom onset. **a** Sagittal T2-weighted image showing no signal abnormalities of the spinal cord. **b** Sagittal and **c** axial T1-weighted images after administration of gadolinium-based contrast agent revealing smooth contrast enhancement of the conus medullaris and the anterior and posterior roots of the cauda equina (arrows) extending beyond the spinal canal into the lumbosacral nerve roots. All MR imaging was performed on a clinical 1.5 T MR scanner (Achieva, Philips Healthcare, Best, the Netherlands) and included a T1-weighted spin-echo sequence (405/7 ms [repetition time (TR)/echo time (TE)] before and after administration of a Gd-DO3A-butrol-based contrast agent (Gadovist, Bayer, Leverkusen, Germany) in axial and sagittal orientation, a T2-weighted turbo spin-echo sequence [2800/90 ms (TR/TE)] as well as a sagittal T2-weighted modified Dixon (mDixon) spin-echo sequence [2800/90 ms (TR/TE)]

The CSF showed no abnormalities. Because the neurophysiological examination detected no F-waves in the lower extremities, a GBS was assumed. A therapy with IVIG was initiated. The symptoms were unaffected by the therapy and persisted. Therefore, the spinal MRI was repeated a month after initial admission. It revealed bilateral circular foci of high T2-weighted signal in the anterior portion of the spinal cord at the L1 level, suggestive of a mature infarction (Fig. 2). Furthermore, two thoracic vertebral bodies showed signs of ischemia (Fig. 2). The diagnosis of a GBS was dismissed and a spinal cord ischemia was diagnosed. Screening for thrombophilia or vasculitis, as well as computer tomography of the Aorta, electro cardiography and transesophageal echocardiography were unobtrusive. A repeated CSF analysis showed no abnormalities. Therefore, the etiology remained unknown.

**Fig. 2:**
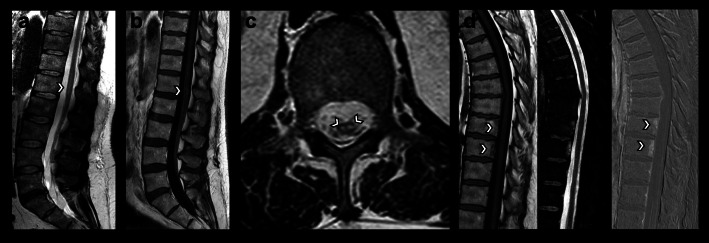
Contrast-enhanced follow-up MRI performed 4 weeks after symptom onset. **a** Sagittal T2-weighted image showing vertical linear foci of high T2-weighted signal located anteriorly within the lower spinal cord (arrow). **b** Sagittal T1-weighted image after contrast injection demonstrating newly occurred intramedullary contrast enhancement (arrow) and persisting enhancement of the conus medullaris and descending nerve roots. **c** Axial T2-weighted image revealing bilateral circular foci of high T2-weighted signal in the anterior portion of the spinal cord at L1 level (arrows), also referred to as owl-eyes sign, suggestive of a mature infarction. **d** Sagittal T1-weighted image without contrast mean (left), sagittal T2-weighted fat-suppressed image (middle) and sagittal T1-weighted subtraction image after gadolinium-based contrast mean injection subtracted by the native T1-weighted image showing focal oedema in the posterior portion of the Th9 and Th10 vertebral bodies with associated contrast uptake, suggestive of vertebral body infarction

The patient was discharged with a secondary prophylaxis of 100 mg acetylsalicylic acid once a day.

Over time and with rehabilitation the patient showed progress in motor recovery and regained walking function, but a permanent catheter was needed, and he had a persistent loss of anal sphincter control.

At first a GBS was assumed due to the extended contrast enhancement as well as the areflexia and loss of F-waves. There are also reports of contrast enhancement in the cauda equina in GBS [9]. After follow up MRI results the diagnosis was revised. The infarction as well as the contrast enhancement stretched from the conus medullaris up to lumbosacral nerve roots and therefore explains the missing F-waves and the areflexia. Due to the spinal lesion no motor or sensible evoked potentials were measurable.

By means of the MRI results we postulate multifocal spinal cord ischemia. First an infarction to the conus medullaris and cauda equina which showed high contrast enhancement and persisted in the follow up examination. Secondly an ischemia to the myelon at L1 with a typical owl-eye sign (Fig. 2). The infarct enhancement may differ on MRI but is best visible some days after infarction [1]. Therefore the spinal cord ischemia was not detected on the first MRI.

With this case in mind, a DWI, which is not routinely performed in the setting of clinically unclear paraparesis at our institution, would probably have been a valuable addition to the routine imaging protocol, and, when performed initially, might have been able to detect and confirm the underlying ischemia. DWI has been widely used for the evaluation of a variety of brain disorders, especially for acute stroke, and previous data suggest that DWI has the potential to be useful in the early detection of spinal infarction. However, the absence of diffusion restriction does not necessarily exclude spinal cord ischemia [10].

Only few reports with similar findings have been published, only 8% of spinal ischemia are associated with nerve root enhancement [10]. Our case is also remarkable, because the clinical presentation and neurophysiological findings mimicked a GBS. Clinicians should keep in mind that spinal infarction may be the underpinning etiology in those cases. Additionally, in cases with acute symptom onset an ischemic lesion should be considered, and a second time-delayed MRI should be performed to detect potential infarction to the spinal cord.

## Data Availability

Data sharing is not applicable to this article as no datasets were generated or analysed during the current study.

## Abbreviations

CSF: Cerebrospinal fluid
DWI: Diffusion-weighted MR imaging
GBS: Guillain-Barré-Syndrome
IVIG: Intravenous immunoglobulin
MRC: Muscle Power Assessment
MRI: Magnetic resonance imaging
